# 1287. Assessing the Utility of Vertebral Body / Disc Space Fluid Cell Differential in the Diagnosis of Native Vertebral Osteomyelitis

**DOI:** 10.1093/ofid/ofad500.1126

**Published:** 2023-11-27

**Authors:** Said El Zein, Madeline Kingsbury, Brian Lahr, Carrie Carr, Felix Diehn, Brett Freedman, Pedro Horna, Matthew Howard, Aaron J Tande, Elie Berbari

**Affiliations:** Mayo Clinic, Rochester, Minnesota; Mayo Clinic, Rochester, Minnesota; Mayo Clinic, Rochester, Minnesota; Mayo Clinic, Rochester, Minnesota; Mayo Clinic, Rochester, Minnesota; Mayo Clinic, Rochester, Minnesota; Mayo Clinic, Rochester, Minnesota; Mayo Clinic, Rochester, Minnesota; Mayo Clinic, Rochester, Minnesota; Mayo Clinic, Rochester, Minnesota

## Abstract

**Background:**

Current approaches to diagnosing suspected native vertebral osteomyelitis (NVO) rely on imaging and microbiological workup, including blood and CT-guided biopsy cultures. These existing diagnostic methods often result in ambiguity, necessitating additional tests to distinguish NVO from its mimics. Our study aims to validate pilot findings demonstrating that an elevated neutrophil differential (PMN) in vertebral bone biopsy/ disc space fluid aspirate is associated with NVO. This may allow stratification of patients with suspected NVO into those who can be monitored closely versus those who may benefit from further invasive testing or empiric antibiotic therapy (Fig 1).

**Figure 1:** Proposed workup of patients with suspected native vertebral osteomyelitis guided by disc space/ vertebral body neutrophil (PMN) differential.

**Figure:**
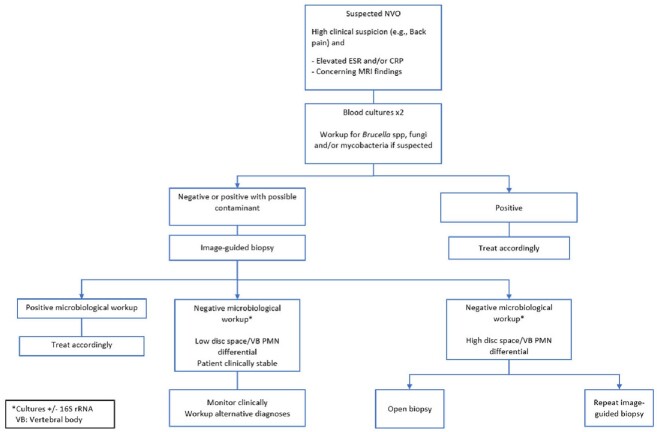

**Methods:**

This analysis examines a subset of patients from a prospective cohort study, enrolling adults referred to Mayo Clinic's neuroradiology department for spine biopsy. After collecting routine clinical specimens, the needle is rinsed with saline into an EDTA tube for automated cell count and differential analysis. To evaluate the predictive capacity of neutrophil (PMN) differential for NVO, logistic regression models were employed, and receiver operating characteristic (ROC) curve analyses were conducted for various PMN percentage cutoff points.

**Results:**

In this preliminary analysis, 39 patients were included, comprising 7 patients with NVO and 32 patients with alternative diagnoses. Baseline characteristics are presented in Table 1. All NVO patients received antibiotics within two weeks of the spine biopsy. The median biopsy sample PMN percentage for NVO patients was 83.0 (77.5-88.0), compared to 65.0 (56.5-69.5) for those without NVO (p=0.005) (Fig 2A). The estimated relationship between the probability of NVO and each measure across the full range of percentages is illustrated in Fig 2B. The optimal ROC curve point corresponds to a PMN differential of 72.5% (sensitivity=0.86, specificity=0.81) (Fig 3).

**Table 1:**
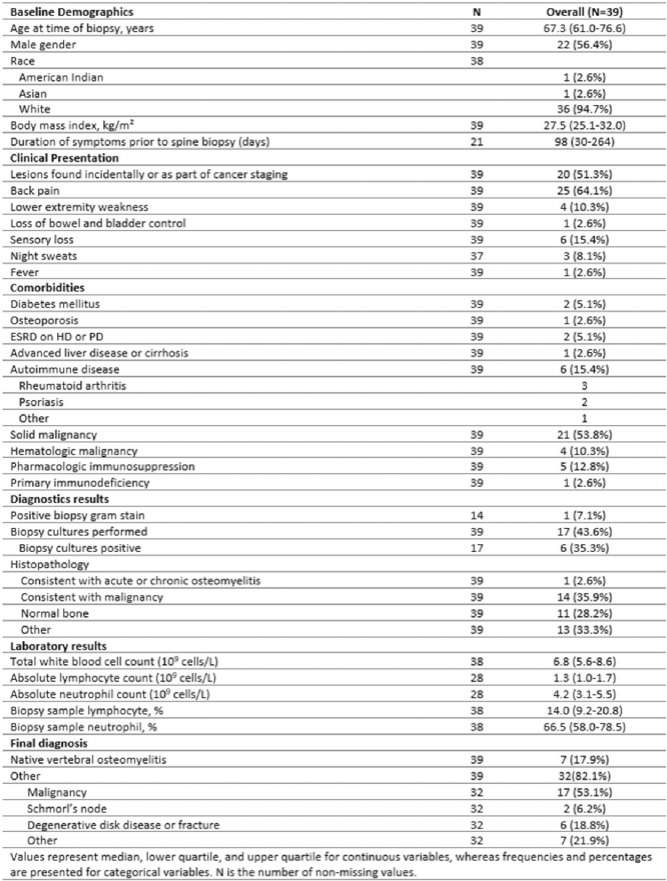
Baseline Characteristics of Patients Included in the Preliminary Analysis

**Figure 2:**
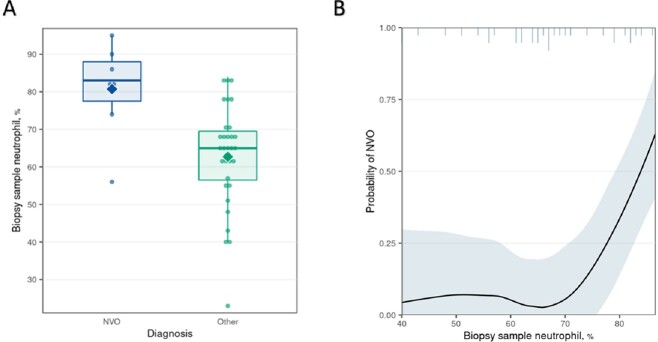
A) Distribution of spine biopsy neutrophil percent by native vertebral osteomyelitis (NVO) status, B) Loess nonparametric smoother showing the relationship between the probability of NVO and biopsy sample neutrophil percentage.

**Figure 3:**
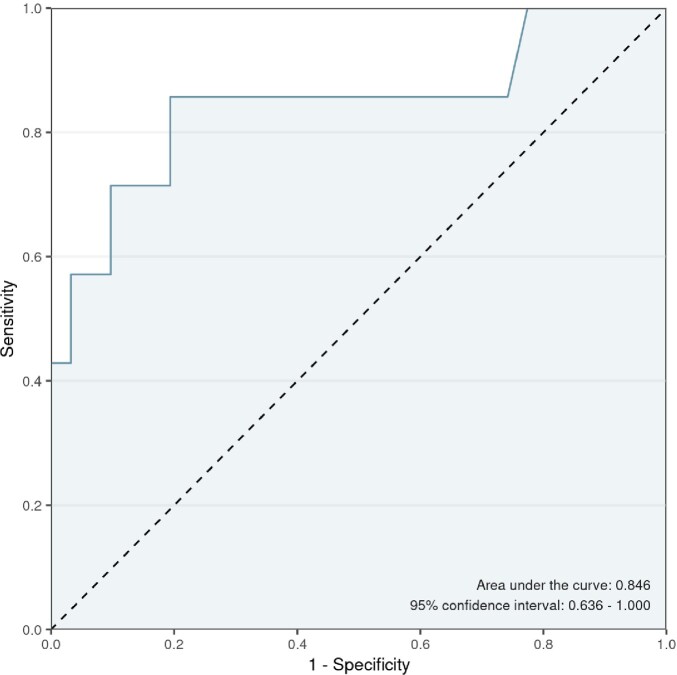
ROC curve analysis of neutrophil percent and NVO

**Conclusion:**

This preliminary analysis indicates that an elevated PMN differential in spine biopsy samples may serve as a valuable diagnostic tool for NVO, with results seemingly unaffected by recent antibiotic intake. The PMN differential cutoff of 72.5% exhibited notable sensitivity and specificity. Investigations are ongoing to further validate these findings

**Disclosures:**

**Brett Freedman, MD**, Clear Choice Therapeutics: Stocks/Bonds|Medtronic: Grant/Research Support|Neuroinnovations: Stocks/Bonds **Aaron J. Tande, M.D.**, Musculoskeletal Infection Society: Board Member|Wolters Kluwer Health - Lippincott Williams & Wilkins: Publishing royalties, financial or material support

